# Supplementary material for: Omega-3 Polyunsaturated Fatty Acids Provoke Apoptosis in Hepatocellular Carcinoma through Knocking Down the STAT3 Activated Signaling Pathway: In Vivo and In Vitro Study
Source: Molecules. 2022 May 9;27(9):3032. doi: 10.3390/molecules27093032 (PMC9103886; doi:10.3390/molecules27093032)
Supplement: Supplementary file 1 [file molecules-27-03032-s001.zip › Supplementary File.pdf]

Supplementary file

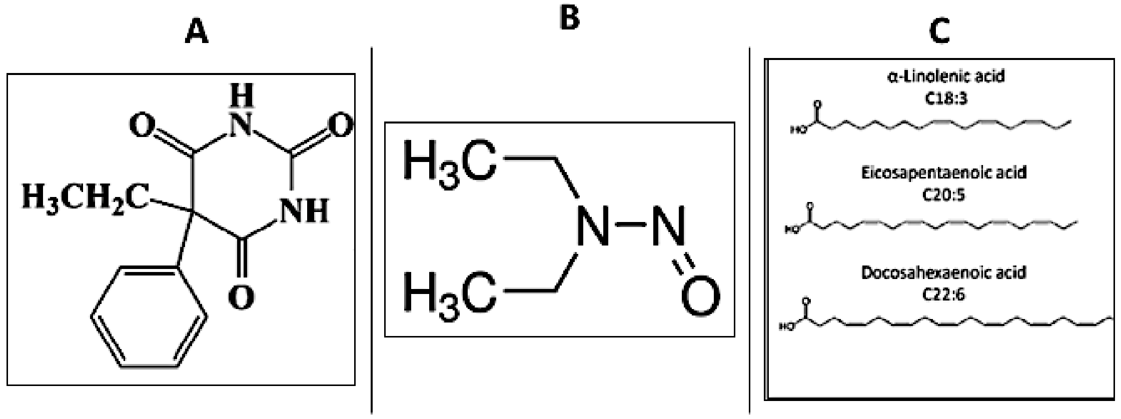

Figure S1. Chemical structure of N-Nitrosodiethylamine (A), phenobarbital (B), and n-3 Polyunsaturated fatty acids (C).

Table S1. Area % of pSTAT3, Cyclin D1, and Bcl-2 expression in different groups as mean ± SD.

***P*-

Groups	Bcl-2	Cyclin D	pSTAT3	F- Value	*P*-Value
Control	29.21 ± 2.744^{a}	4.223 ± 1.496^{a}	7.567 ± 2.512^{a}	680.755	0.000**
DEN	83.326 ± 3.529^{c}	79.324 ± 2.452^{c}	71.547 ± 2.657^{c}	2553.082	0.000**
n-3 PUFAs	40.564 ± 1.244^{b}	14.024 ± 1.87^{b}	25.303 ± 3.326^{b}	803.925	0.000**

Values ≤ 0.001 are highly significant.

Different superscripts indicate significant difference at $p \leq 0.05$.

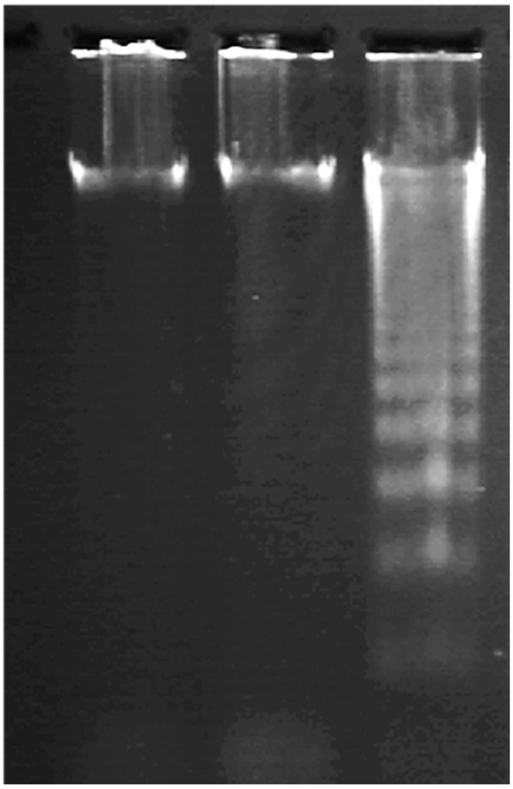

Figure S2. Raw gel image of the influence of n-3 PUFAs on DEN-induced hepatic DNA

Figure S3A

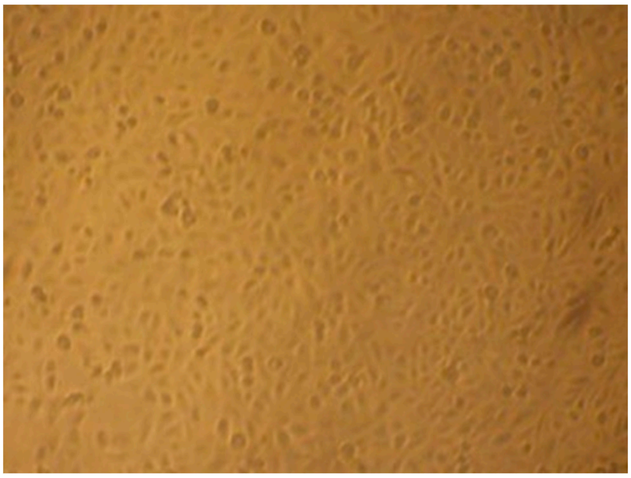

Figure S3B

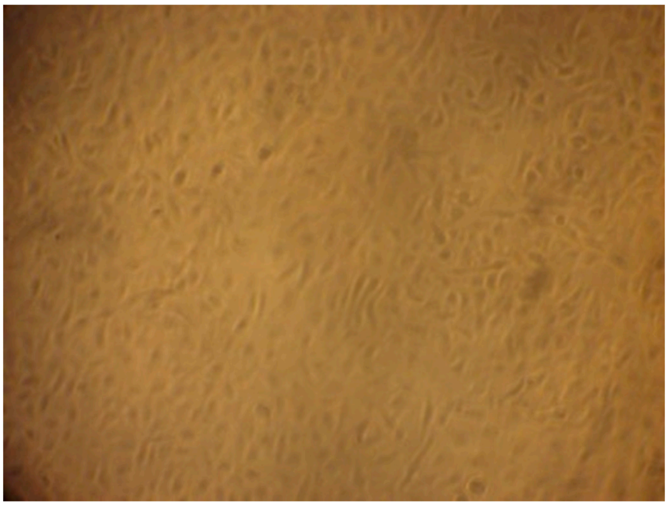

Figure S3C

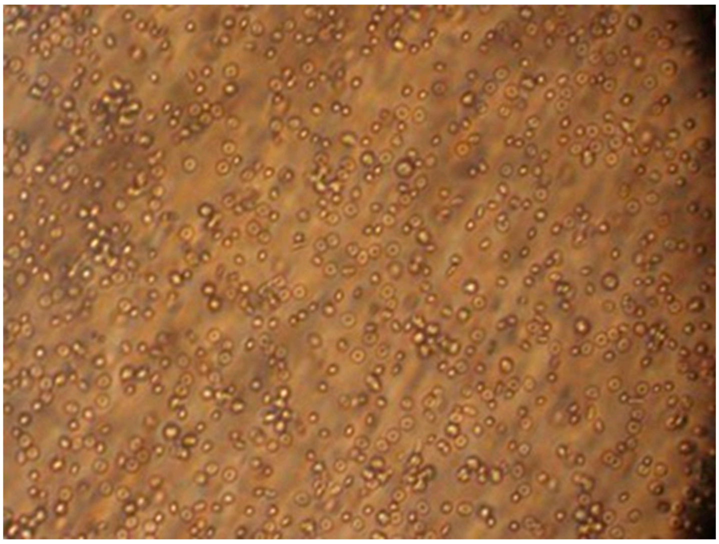

Figure S3 (A,B,C). Original images for the morphological changes of HepG2 cells at 40×10 magnifications

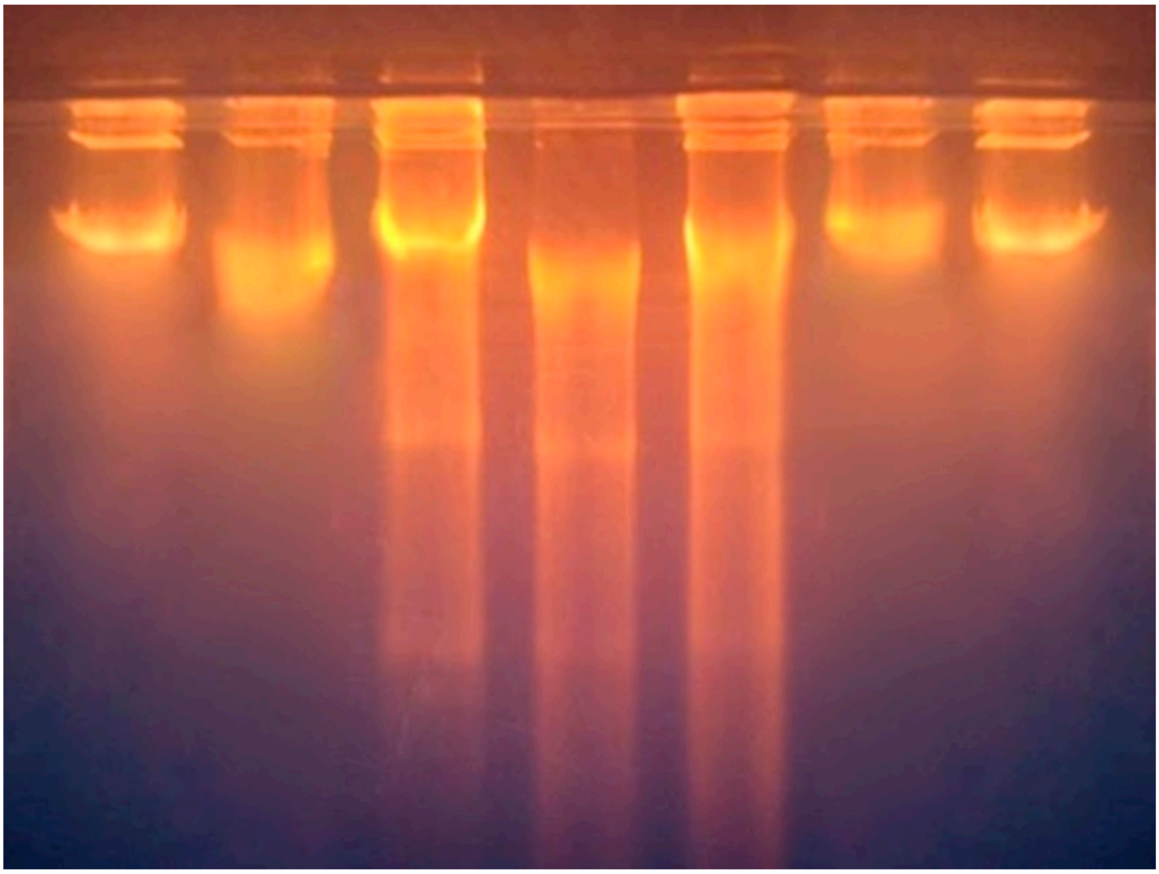

Figure S4. Raw gel image for the detection of DNA fragmentation by agarose gel electrophoresis

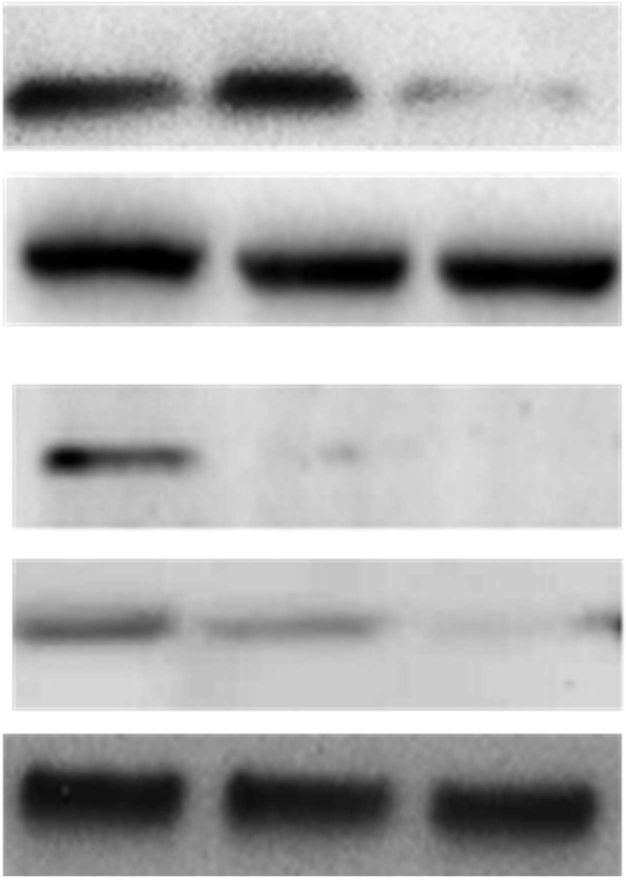

Figure S5. Western blot image for p-STAT3, STAT3, Bcl-2, Cyclin D1 and β-actin protein
